# *eNeuro* Assessments: A Standardized Way to Communicate Research Quality

**DOI:** 10.1523/ENEURO.0310-26.2026

**Published:** 2026-10-20

**Authors:** Mihaela D. Iordanova

**Affiliations:** Deputy Editor-in-Chief, *eNeuro* Center for Studies in Behavioural Neurobiology, Department of Psychology, Concordia University, Montreal, Quebec, Canada

One of the most enduring mantras in science is “publish or perish.” However, it is not enough merely to publish; where one publishes matters. Employment, tenure, promotion, grant funding, and awards not only depend on the quantity of papers produced but also on the reputation of the journals in which the papers appear. High-profile journals (i.e., journals with a high impact factor) influence research visibility, commanding the attention of scientists across disciplines and attract interest from policymakers, journalists, and the public. Publication in such journals signals that a paper is significant, with the potential to shape future research. As a result, journal reputation has become a widely used proxy for research quality; shaping careers, funding, and research directions (McKiernan et al., 2019). However, important and influential research appears across a wide range of journals (Bartlett et al., 2024). Although simple and convenient, a journal's impact factor only reflects average citation patterns across its articles; it neither explains what makes an individual paper valuable nor reliably captures the many ways scientific work contributes to progress: novel ideas, theoretical breakthroughs, methodological or analytical advances, valuable datasets, or rigorous evidence. The challenge, therefore, is to give decision-makers a reliable shorthand for evaluating individual contributions on their own merits.

To help shift attention from journal-level metrics to the merits of individual papers, *eNeuro* is adopting an innovative method for publicly sharing editorial evaluations, termed ***eNeuro* Assessments**. These assessments, consisting of short statements developed collaboratively by expert reviewers and editors, are designed to communicate how a manuscript was evaluated during peer review in a transparent and standardized manner. Using a common vocabulary adapted from the framework implemented at *eLife* (https://elifesciences.org/about/elife-assessments), each assessment will summarize how a paper was evaluated along two dimensions commonly considered when judging scientific contributions: the significance of the findings and the quality of the data supporting them. Every manuscript published in *eNeuro* will soon be accompanied by an *eNeuro* Assessment, which will be displayed immediately below the abstract in lieu of our traditional author-written significance statements.

How will the process work in practice? Upon submission to *eNeuro*, each manuscript is assigned to a Reviewing Editor whose expertise aligns with the scientific content of the paper. Expert reviewers, including both Associate Reviewers (Iordanova, 2026) and ad hoc reviewers, are then invited to evaluate the manuscript. In addition to traditional peer-review feedback, we will now ask the reviewers to explicitly comment on the significance of the findings and the quality of the data using the terms outlined in Table 1. The vocabulary uses positive language to recognize the diverse ways research advances science, valuing both broadly influential discoveries and studies with a more focused scope. Once all reviews have been submitted, the reviewers, Reviewing Editor, and an Advisory Board Member engage in a consultation process to discuss the manuscript and reconcile differences in opinion. Based on these discussions, a review synthesis is prepared that summarizes the major strengths, limitations, and recommendations arising from peer review. At the same time, the participants will collectively determine the editorial assessment, including which standardized descriptors best suit the work. Both the review synthesis and the provisional editorial assessment are communicated to the authors as part of the decision letter. Following revision and resubmission, the manuscript and authors' responses are re-evaluated, and the editorial assessment is updated as necessary to reflect the final version of the paper. The final *eNeuro* Assessment will appear directly below each article's abstract, with its standardized descriptors shown in **bold**. Because peer reviews and decision letters are published alongside each article, *eNeuro* Assessments are fully transparent.

**Table 1.** Common vocabulary of assessment terms adapted from the framework implemented at *elife* (Bartlett et al., 2024)

Significance of findings	Quality of the data
**Landmark:** Findings with profound implications and widespread influence, which are likely to be of broad interest	**Exceptional:** Exemplary use of existing and new methods that establish new standards for a field
**Fundamental:** Findings that substantially advance understanding of important research questions	**Compelling:** High-quality data and analyses, more rigorous than the current state-of-the-art
**Important:** Findings with theoretical or practical implications for multiple subfields	**Convincing:** Appropriate and validated methodology in line with current state-of-the-art, with good support for the claims
**Valuable:** Findings with theoretical or practical implications for a subfield	**Solid:** Uses appropriate methodology, with minor weaknesses
**Useful:** Findings with focused importance and scope	**Incomplete:** Methodology provides some support for the main claims with some limitations
**Not Significant:** Findings that do not make a notable impact	**Inadequate:** Methodology does not provide support for the primary claims

By explicitly evaluating the significance of the findings and the strength of the supporting evidence, the assessments will help readers understand both a paper's contribution and the confidence warranted by its conclusions. Moreover, by situating each study within its broader scientific context, *eNeuro* Assessments provide authors with a more informative, transparent, and credible description of their research impact that can be shared with hiring, funding, promotion, and award committees. With this initiative, *eNeuro* moves scientific evaluation toward transparent and standardized assessments that reflect the added value of research itself. Our hope is that an *eNeuro* Assessment will have meaning not only within the *eNeuro* ecosystem but also more broadly across neuroscience and that this will in turn encourage other journals to adopt similar initiatives for the benefit of all.
